# Supplementary material for: End Sequence Analysis Toolkit (ESAT) expands the extractable information from single-cell RNA-seq data
Source: Genome Res. 2016 Oct;26(10):1397–410. doi: 10.1101/gr.207902.116 (PMC5052061; doi:10.1101/gr.207902.116)
Supplement: Supplemental Material [file supp_gr.207902.116_Supp_Materials.tar.gz › ESAT-esat_v0.1/doc/html/interfaceumms_1_1core_1_1annotation_1_1_annotation.html]

ESAT: umms.core.annotation.Annotation Interface Reference


ESAT


- Main Page
- Related Pages
- Packages
- Classes
- Files

- Class List
- Class Index
- Class Hierarchy
- Class Members

All Classes Namespaces Files Functions Variables Enumerator Pages

- umms
- core
- annotation
- Annotation

Classes |
Public Member Functions |
List of all members

umms.core.annotation.Annotation Interface Reference

Inheritance diagram for umms.core.annotation.Annotation:

[legend]

Collaboration diagram for umms.core.annotation.Annotation:

[legend]

|  |  |
| --- | --- |
| Classes | |
| enum | Strand |
|  | |

|  |  |
| --- | --- |
| Public Member Functions | |
| int | getStart () |
|  | |
| int | getSAMStart () |
|  | |
| int | getEnd () |
|  | |
| int | getSAMEnd () |
|  | |
| String | getReferenceName () |
|  | |
| String | getChr () |
|  | |
| String | getName () |
|  | |
| Strand | getOrientation () |
|  | |
| Strand | getStrand () |
|  | |
| boolean | hasOrientation () |
|  | |
| boolean | isNegativeStrand () |
|  | |
| int | numBlocks () |
|  | |
| List<?extends Annotation > | getBlocks () |
|  | |
| List<?extends Annotation > | getBlocks (boolean oriented) |
|  | |
| int | length () |
|  | |
| int | getSize () |
|  | |
| int | size () |
|  | |
| int | getLengthOnReference () |
|  | |
| int | getOrientedStart () |
|  | |
| int | getOrientedEnd () |
|  | |
| double | getScore () |
|  | |
| boolean | isUnoriented () |
|  | |
| int | getReferenceCoordinateAtPosition (int positionInAnnotation) |
|  | |
| int | getPositionAtReferenceCoordinate (int referenceCoordinate) |
|  | |
| int | getReferenceCoordinateAtPosition (int positionInAnnotation, boolean ignoreOrientation) |
|  | |
| int | getPositionAtReferenceCoordinate (int referenceCoordinate, boolean ignoreOrientation) |
|  | |
| void | setStart (int start) |
|  | |
| void | setEnd (int end) |
|  | |
| void | setOrientation (char orientation) |
|  | |
| void | setOrientation (Strand orientation) |
|  | |
| void | setOrientedStart (int orientedStart) |
|  | |
| void | setOrientedEnd (int orientedEnd) |
|  | |
| void | setReferenceName (String refName) |
|  | |
| void | setName (String name) |
|  | |
| void | setScore (double score) |
|  | |
| boolean | equals (Annotation other) |
|  | |
| void | expand (int deltaStart, int deltaEnd) |
|  | |
| Annotation | trim (int deltaStart, int deltaEnd) |
|  | |
| void | shift (int delta) |
|  | |
| void | moveToCoordinate (int coordinateInReference) |
|  | |
| Annotation | copy () |
|  | |
| List< Annotation > | disect (Annotation a) |
|  | |
| List< Annotation > | disect (List<?extends Annotation > disectors) |
|  | |
| Annotation | minus (Annotation other) |
|  | |
| Annotation | minus (Collection<?extends Annotation > others) |
|  | |
| int | getDistanceTo (Annotation other) |
|  | |
| String | toUCSC () |
|  | |
| String | toBED () |
|  | |
| String | toBED (int r, int g, int b) |
|  | |
| String | toShortBED () |
|  | |
| String | toBEDGraph () |
|  | |
| String | getFullInfoString () |
|  | |
| boolean | overlaps (Annotation other, int buffer) |
|  | |
| boolean | overlaps (Collection<?extends Annotation > others, int buffer) |
|  | |
| boolean | overlaps (Annotation other) |
|  | |
| boolean | overlaps (Collection<?extends Annotation > others) |
|  | |
| boolean | overlaps (Annotation other, int buffer, boolean considerOrientation) |
|  | |
| boolean | overlaps (Annotation other, boolean considerOrientation) |
|  | |
| boolean | overlapsStranded (Annotation other) |
|  | |
| int | getOverlap (Annotation other) |
|  | |
| boolean | contains (Annotation other) |
|  | |
| Annotation | union (Annotation other) |
|  | |
| Annotation | intersect (Annotation other) |
|  | |
| List< Annotation > | intersect (List<?extends Annotation > annotations) |
|  | |
| int | compareToAnnotation (Annotation b) |
|  | |
| void | stitchTo (Annotation next) |
|  | |
| String | toCigar () |
|  | |
| boolean | fullyContains (Annotation annotation) |
|  | |
| Annotation | complement () |
|  | |
| boolean | equals (Annotation other, boolean useOrientation) |
|  | |
| Collection<?extends Annotation > | getSpliceConnections () |
|  | |
| int | getMidpoint () |
|  | |

## Member Function Documentation

|  |  |  |  |  |  |
| --- | --- | --- | --- | --- | --- |
| int umms.core.annotation.Annotation.compareToAnnotation | ( | Annotation | *b* | ) |  |

Should behave like compareTo for a basic annotation

Parameters
:   |  |  |
    | --- | --- |
    | b |  |

Returns

Implemented in umms.core.annotation.AbstractAnnotation.

|  |  |  |  |  |
| --- | --- | --- | --- | --- |
| Annotation umms.core.annotation.Annotation.complement | ( |  | ) |  |

Returns
:   a new CompoundInterval containing the gaps between the blocks in this annotation

Implemented in umms.core.annotation.BasicAnnotation.

|  |  |  |  |  |  |
| --- | --- | --- | --- | --- | --- |
| boolean umms.core.annotation.Annotation.contains | ( | Annotation | *other* | ) |  |

Parameters
:   |  |  |
    | --- | --- |
    | other |  |

Returns
:   true if the blocks of other are fully contained within the blocks of this object

Implemented in umms.core.annotation.BasicAnnotation.

Here is the caller graph for this function:

|  |  |  |  |  |
| --- | --- | --- | --- | --- |
| Annotation umms.core.annotation.Annotation.copy | ( |  | ) |  |

Returns
:   A deep clone of this Annotation object

Implemented in umms.core.annotation.Gene, and umms.core.annotation.BasicAnnotation.

Here is the caller graph for this function:

|  |  |  |  |  |  |
| --- | --- | --- | --- | --- | --- |
| List<Annotation> umms.core.annotation.Annotation.disect | ( | Annotation | *a* | ) |  |

Fragments the current annotation if it overlaps the provided one. it returns an empty list if this annotation does not overlap the one passed in.

Parameters
:   |  |  |
    | --- | --- |
    | Annotation | annotation to disect the current one |

Returns
:   List<Annotation> of the disected annotations, an empty list is returned if the annotations do not overlap.

Implemented in umms.core.annotation.BasicAnnotation, and broad.core.annotation.BasicGenomicAnnotation.

|  |  |  |  |  |  |
| --- | --- | --- | --- | --- | --- |
| List<Annotation> umms.core.annotation.Annotation.disect | ( | List<?extends Annotation > | *disectors* | ) |  |

Fragments the current annotation if it overlaps the provided ones. It returns a list with one component (this annotation) if no annotation in the provided list overlaps the discted annotaion.

Parameters
:   |  |  |
    | --- | --- |
    | List<GenomicAnnotation> | **sorted** annotations with which to disect the current one. |

Returns
:   List<GenomicAnnotation> of the disected annotations, a list just this annotation is returned if the annotations do not overlap.

Implemented in umms.core.annotation.BasicAnnotation, and broad.core.annotation.BasicGenomicAnnotation.

|  |  |  |  |  |  |
| --- | --- | --- | --- | --- | --- |
| boolean umms.core.annotation.Annotation.equals | ( | Annotation | *other* | ) |  |

Implemented in umms.core.alignment.SingleEndAlignment, and umms.core.annotation.AbstractAnnotation.

Here is the caller graph for this function:

|  |  |  |  |
| --- | --- | --- | --- |
| boolean umms.core.annotation.Annotation.equals | ( | Annotation | *other*, |
|  |  | boolean | *useOrientation* |
|  | ) |  |  |

Test whether two annotations are equal regardless of whether the orientation are the same

Parameters
:   |  |  |
    | --- | --- |
    | other | The annotation to test equality to |
    | useOrientation | true=ensure strands match false=OK if strands dont match |

Returns

Implemented in umms.core.annotation.AbstractAnnotation.

|  |  |  |  |
| --- | --- | --- | --- |
| void umms.core.annotation.Annotation.expand | ( | int | *deltaStart*, |
|  |  | int | *deltaEnd* |
|  | ) |  |  |

Expand annotation by a set number of bases, taking into account blocks

Parameters
:   |  |  |
    | --- | --- |
    | deltaStart | positive extends the gene. |
    | deltaEnd | positive extends the gene. |

Implemented in umms.core.annotation.AbstractAnnotation.

|  |  |  |  |  |  |
| --- | --- | --- | --- | --- | --- |
| boolean umms.core.annotation.Annotation.fullyContains | ( | Annotation | *annotation* | ) |  |

Test whether the annotation is fully contained in this

Parameters
:   |  |  |
    | --- | --- |
    | annotation |  |

Returns

Implemented in umms.core.annotation.AbstractAnnotation.

|  |  |  |  |  |
| --- | --- | --- | --- | --- |
| List<? extends Annotation> umms.core.annotation.Annotation.getBlocks | ( |  | ) |  |

Returns
:   list of blocks in coordinate order

Implemented in umms.core.annotation.BasicAnnotation, broad.core.annotation.BED, and broad.core.annotation.GenomicAnnotation.

Here is the caller graph for this function:

|  |  |  |  |  |  |
| --- | --- | --- | --- | --- | --- |
| List<? extends Annotation> umms.core.annotation.Annotation.getBlocks | ( | boolean | *oriented* | ) |  |

Parameters
:   |  |  |
    | --- | --- |
    | oriented | set to true if the list should be ordered according to the annotation orientation and by position |

Returns

Implemented in umms.core.annotation.BasicAnnotation.

|  |  |  |  |  |
| --- | --- | --- | --- | --- |
| String umms.core.annotation.Annotation.getChr | ( |  | ) |  |

Returns
:   name of the reference sequence

Implemented in umms.core.alignment.AbstractPairedEndAlignment, umms.core.alignment.SingleEndAlignment, umms.core.alignment.Alignment, and umms.core.annotation.AbstractAnnotation.

Here is the caller graph for this function:

|  |  |  |  |  |  |
| --- | --- | --- | --- | --- | --- |
| int umms.core.annotation.Annotation.getDistanceTo | ( | Annotation | *other* | ) |  |

Calculates the distance to the another genomic annotation.

Returns
:   0 if the annotations overlap or the minimum between the edges otherwise.

Implemented in umms.core.annotation.AbstractAnnotation.

|  |  |  |  |  |
| --- | --- | --- | --- | --- |
| int umms.core.annotation.Annotation.getEnd | ( |  | ) |  |

Returns
:   end of the annotation (exclusive)

Implemented in umms.core.alignment.AbstractPairedEndAlignment, umms.core.annotation.BasicAnnotation, umms.core.alignment.Alignment, and broad.core.annotation.LightweightGenomicAnnotation.

Here is the caller graph for this function:

|  |  |  |  |  |
| --- | --- | --- | --- | --- |
| String umms.core.annotation.Annotation.getFullInfoString | ( |  | ) |  |

Returns
:   A string with no whitespace that includes all information about the annotation

Implemented in umms.core.annotation.AbstractAnnotation.

|  |  |  |  |  |
| --- | --- | --- | --- | --- |
| int umms.core.annotation.Annotation.getLengthOnReference | ( |  | ) |  |

Returns
:   The distance between the start of the first block and end of the last on the reference sequence (including spaces between blocks)

Implemented in umms.core.annotation.AbstractAnnotation.

Here is the caller graph for this function:

|  |  |  |  |  |
| --- | --- | --- | --- | --- |
| int umms.core.annotation.Annotation.getMidpoint | ( |  | ) |  |

This will return the position in the center of this annotation taking into account the blocked structure

Returns

Implemented in umms.core.annotation.BasicAnnotation, and umms.core.annotation.AbstractAnnotation.

|  |  |  |  |  |
| --- | --- | --- | --- | --- |
| String umms.core.annotation.Annotation.getName | ( |  | ) |  |

Returns
:   the name of the annotation

Implemented in umms.core.annotation.BasicAnnotation, and broad.core.annotation.LightweightGenomicAnnotation.

Here is the caller graph for this function:

|  |  |  |  |  |
| --- | --- | --- | --- | --- |
| Strand umms.core.annotation.Annotation.getOrientation | ( |  | ) |  |

Returns
:   orientation or strand

Implemented in umms.core.alignment.AbstractPairedEndAlignment, umms.core.annotation.BasicAnnotation, broad.core.annotation.LightweightGenomicAnnotation, and broad.core.annotation.GenomicAnnotation.

Here is the caller graph for this function:

|  |  |  |  |  |
| --- | --- | --- | --- | --- |
| int umms.core.annotation.Annotation.getOrientedEnd | ( |  | ) |  |

Returns
:   the 3-prime end of the annotation, considering its orientation or strand

Implemented in broad.core.annotation.BasicGenomicAnnotation, umms.core.annotation.AbstractAnnotation, and broad.core.annotation.GenomicAnnotation.

|  |  |  |  |  |
| --- | --- | --- | --- | --- |
| int umms.core.annotation.Annotation.getOrientedStart | ( |  | ) |  |

Returns
:   the 5-prime end of the annotation, considering its orientation or strand

Implemented in broad.core.annotation.BasicGenomicAnnotation, umms.core.annotation.AbstractAnnotation, and broad.core.annotation.GenomicAnnotation.

|  |  |  |  |  |  |
| --- | --- | --- | --- | --- | --- |
| int umms.core.annotation.Annotation.getOverlap | ( | Annotation | *other* | ) |  |

Returns the number of bases that overlap between the two annotations.

Parameters
:   |  |  |
    | --- | --- |
    | other |  |

Returns

Implemented in umms.core.annotation.BasicAnnotation.

|  |  |  |  |  |  |
| --- | --- | --- | --- | --- | --- |
| int umms.core.annotation.Annotation.getPositionAtReferenceCoordinate | ( | int | *referenceCoordinate* | ) |  |

Parameters
:   |  |  |
    | --- | --- |
    | referenceCoordinate | 0-based reference coordinate |

Returns
:   0-based position in the Annotation, considering orientation

Implemented in umms.core.annotation.AbstractAnnotation.

|  |  |  |  |
| --- | --- | --- | --- |
| int umms.core.annotation.Annotation.getPositionAtReferenceCoordinate | ( | int | *referenceCoordinate*, |
|  |  | boolean | *ignoreOrientation* |
|  | ) |  |  |

Implemented in umms.core.annotation.BasicAnnotation.

|  |  |  |  |  |  |
| --- | --- | --- | --- | --- | --- |
| int umms.core.annotation.Annotation.getReferenceCoordinateAtPosition | ( | int | *positionInAnnotation* | ) |  |

Parameters
:   |  |  |
    | --- | --- |
    | positionInAnnotation | 0-based position in the Annotation |

Returns
:   0-based reference coordinate at the specified position, considering orientation. NOTE: positionInAnnotation=0 for a negative-strand annotation will match getEnd()

Implemented in umms.core.annotation.AbstractAnnotation.

|  |  |  |  |
| --- | --- | --- | --- |
| int umms.core.annotation.Annotation.getReferenceCoordinateAtPosition | ( | int | *positionInAnnotation*, |
|  |  | boolean | *ignoreOrientation* |
|  | ) |  |  |

Implemented in umms.core.annotation.BasicAnnotation.

|  |  |  |  |  |
| --- | --- | --- | --- | --- |
| String umms.core.annotation.Annotation.getReferenceName | ( |  | ) |  |

Returns
:   name of the reference sequence

Implemented in umms.core.annotation.BasicAnnotation.

Here is the caller graph for this function:

|  |  |  |  |  |
| --- | --- | --- | --- | --- |
| int umms.core.annotation.Annotation.getSAMEnd | ( |  | ) |  |

Returns the start position of this window into SAM coordinate space SAM coordinates are 1-based and inclusive whereas all of our objects are 0-based exclusive

Returns

Implemented in umms.core.annotation.AbstractAnnotation.

|  |  |  |  |  |
| --- | --- | --- | --- | --- |
| int umms.core.annotation.Annotation.getSAMStart | ( |  | ) |  |

Returns the start position of this annotation into SAM coordinate space SAM coordinates are 1-based and inclusive whereas all of our objects are 0-based exclusive

Returns

Implemented in umms.core.annotation.AbstractAnnotation.

Here is the caller graph for this function:

|  |  |  |  |  |
| --- | --- | --- | --- | --- |
| double umms.core.annotation.Annotation.getScore | ( |  | ) |  |

Implemented in umms.core.annotation.BasicAnnotation, broad.core.annotation.BasicLightweightAnnotation, broad.pda.datastructures.Alignments, and broad.core.annotation.LightweightGenomicAnnotation.

Here is the caller graph for this function:

|  |  |  |  |  |
| --- | --- | --- | --- | --- |
| int umms.core.annotation.Annotation.getSize | ( |  | ) |  |

Returns
:   same as size();

Implemented in umms.core.annotation.Gene, umms.core.feature.GeneWindow, and umms.core.annotation.AbstractAnnotation.

Here is the caller graph for this function:

|  |  |  |  |  |
| --- | --- | --- | --- | --- |
| Collection<? extends Annotation> umms.core.annotation.Annotation.getSpliceConnections | ( |  | ) |  |

This will return a collection of splice junctions This is not the same as all gaps, it will only return splice junctions

Returns
:   A collection of splice junctions

Implemented in umms.core.annotation.BasicAnnotation, umms.core.alignment.SingleEndAlignment, and umms.core.alignment.Alignment.

Here is the caller graph for this function:

|  |  |  |  |  |
| --- | --- | --- | --- | --- |
| int umms.core.annotation.Annotation.getStart | ( |  | ) |  |

Returns
:   start of the annotation (inclusive)

Implemented in umms.core.alignment.AbstractPairedEndAlignment, umms.core.annotation.BasicAnnotation, umms.core.alignment.Alignment, and broad.core.annotation.LightweightGenomicAnnotation.

Here is the caller graph for this function:

|  |  |  |  |  |
| --- | --- | --- | --- | --- |
| Strand umms.core.annotation.Annotation.getStrand | ( |  | ) |  |

Returns
:   orientation or strand

Implemented in broad.core.annotation.GFF, and umms.core.annotation.AbstractAnnotation.

Here is the caller graph for this function:

|  |  |  |  |  |
| --- | --- | --- | --- | --- |
| boolean umms.core.annotation.Annotation.hasOrientation | ( |  | ) |  |

Returns
:   true if strand is known

Implemented in umms.core.annotation.AbstractAnnotation.

|  |  |  |  |  |  |
| --- | --- | --- | --- | --- | --- |
| Annotation umms.core.annotation.Annotation.intersect | ( | Annotation | *other* | ) |  |

Returns the result of intersecting this instance with another

Parameters
:   |  |  |
    | --- | --- |
    | other |  |

Returns
:   returns null if there is no intersection

Implemented in umms.core.annotation.BasicAnnotation.

|  |  |  |  |  |  |
| --- | --- | --- | --- | --- | --- |
| List<Annotation> umms.core.annotation.Annotation.intersect | ( | List<?extends Annotation > | *annotations* | ) |  |

Returns the result of intersecting this instance with a list of annotations, with one (possibly blocked) intersection Annotation for each provided annotation

Parameters
:   |  |  |
    | --- | --- |
    | other | annotations |

Returns
:   List of intersections or empty list if none

Implemented in umms.core.annotation.AbstractAnnotation.

|  |  |  |  |  |
| --- | --- | --- | --- | --- |
| boolean umms.core.annotation.Annotation.isNegativeStrand | ( |  | ) |  |

Implemented in umms.core.annotation.Gene, umms.core.alignment.AbstractPairedEndAlignment, umms.core.alignment.Alignment, and umms.core.annotation.AbstractAnnotation.

Here is the caller graph for this function:

|  |  |  |  |  |
| --- | --- | --- | --- | --- |
| boolean umms.core.annotation.Annotation.isUnoriented | ( |  | ) |  |

Returns
:   false if the annotation is not in either negative or positive orientation

Implemented in umms.core.annotation.BasicAnnotation.

Here is the caller graph for this function:

|  |  |  |  |  |
| --- | --- | --- | --- | --- |
| int umms.core.annotation.Annotation.length | ( |  | ) |  |

Returns
:   length of the Annotation; if it has blocks, the gaps between blocks are not included

Implemented in umms.core.annotation.BasicAnnotation, broad.core.annotation.BasicLightweightAnnotation, broad.pda.datastructures.Alignments, and broad.core.annotation.LightweightGenomicAnnotation.

Here is the caller graph for this function:

|  |  |  |  |  |  |
| --- | --- | --- | --- | --- | --- |
| Annotation umms.core.annotation.Annotation.minus | ( | Annotation | *other* | ) |  |

Returns the difference (all regions in this genomic annotation not in the given other annotation) between this genomic annotation and the one

Parameters
:   |  |  |
    | --- | --- |
    | other | - the annotations to take the difference against |

Returns
:   A blocked annotation of the resulting difference.

Implemented in umms.core.annotation.BasicAnnotation.

|  |  |  |  |  |  |
| --- | --- | --- | --- | --- | --- |
| Annotation umms.core.annotation.Annotation.minus | ( | Collection<?extends Annotation > | *others* | ) |  |

Returns the difference (all regions in this genomic annotation not in the given list) between this genomic annotation and the given list

Parameters
:   |  |  |
    | --- | --- |
    | others | - the annotations to take the difference against |

Returns
:   A blocked annotation of the resulting difference.

Implemented in umms.core.annotation.BasicAnnotation.

|  |  |  |  |  |  |
| --- | --- | --- | --- | --- | --- |
| void umms.core.annotation.Annotation.moveToCoordinate | ( | int | *coordinateInReference* | ) |  |

Move an annotation, preserving the relationships between its blocks, to a new coordinate.

Parameters
:   |  |  |
    | --- | --- |
    | coordinateInReference |  |

Implemented in umms.core.annotation.BasicAnnotation, and umms.core.alignment.AbstractPairedEndAlignment.

|  |  |  |  |  |
| --- | --- | --- | --- | --- |
| int umms.core.annotation.Annotation.numBlocks | ( |  | ) |  |

Returns
:   number of blocks in the Annotation

Implemented in umms.core.annotation.BasicAnnotation.

Here is the caller graph for this function:

|  |  |  |  |
| --- | --- | --- | --- |
| boolean umms.core.annotation.Annotation.overlaps | ( | Annotation | *other*, |
|  |  | int | *buffer* |
|  | ) |  |  |

Parameters
:   |  |  |
    | --- | --- |
    | other | - other genomic annotation |
    | buffer | if the overlap is within buffer they will be considered overlapping even if they do not overlap within their original boundaries. |

Returns
:   true if they overlap in this extended definition, not considering orientation

Implemented in umms.core.annotation.AbstractAnnotation.

Here is the caller graph for this function:

|  |  |  |  |
| --- | --- | --- | --- |
| boolean umms.core.annotation.Annotation.overlaps | ( | Collection<?extends Annotation > | *others*, |
|  |  | int | *buffer* |
|  | ) |  |  |

Parameters
:   |  |  |
    | --- | --- |
    | other | - other genomic annotation |
    | buffer | if the overlap is within buffer they will be considered overlapping even if they do not overlap within their original boundaries. |

Returns
:   true if they overlap in this extended definition, not considering orientation

Implemented in broad.core.annotation.BasicLightweightAnnotation, and umms.core.annotation.AbstractAnnotation.

|  |  |  |  |  |  |
| --- | --- | --- | --- | --- | --- |
| boolean umms.core.annotation.Annotation.overlaps | ( | Annotation | *other* | ) |  |

Parameters
:   |  |  |
    | --- | --- |
    | other | GenomicAnnotation to check of overlap |

Returns
:   true if the current instance overlaps with the other one, not considering orientation

Implemented in umms.core.annotation.AbstractAnnotation.

|  |  |  |  |  |  |
| --- | --- | --- | --- | --- | --- |
| boolean umms.core.annotation.Annotation.overlaps | ( | Collection<?extends Annotation > | *others* | ) |  |

Parameters
:   |  |  |
    | --- | --- |
    | other | GenomicAnnotations to check of overlap |

Returns
:   true if the current instance overlaps with the other one, not considering orientation

Implemented in broad.core.annotation.BasicLightweightAnnotation, and umms.core.annotation.AbstractAnnotation.

|  |  |  |  |
| --- | --- | --- | --- |
| boolean umms.core.annotation.Annotation.overlaps | ( | Annotation | *other*, |
|  |  | int | *buffer*, |
|  |  | boolean | *considerOrientation* |
|  | ) |  |  |

Implemented in umms.core.annotation.BasicAnnotation.

|  |  |  |  |
| --- | --- | --- | --- |
| boolean umms.core.annotation.Annotation.overlaps | ( | Annotation | *other*, |
|  |  | boolean | *considerOrientation* |
|  | ) |  |  |

Implemented in umms.core.annotation.AbstractAnnotation.

|  |  |  |  |  |  |
| --- | --- | --- | --- | --- | --- |
| boolean umms.core.annotation.Annotation.overlapsStranded | ( | Annotation | *other* | ) |  |

Implemented in umms.core.annotation.AbstractAnnotation.

|  |  |  |  |  |  |
| --- | --- | --- | --- | --- | --- |
| void umms.core.annotation.Annotation.setEnd | ( | int | *end* | ) |  |

Sets a new end position. Will delete and/or truncate blocks if the new end position is less than the old one. Will extend the last block if the new end position is greater than old one.

Parameters
:   |  |  |
    | --- | --- |
    | new | end position (ignoring orientation) of the annotation |

Implemented in umms.core.annotation.BasicAnnotation, and broad.core.annotation.LightweightGenomicAnnotation.

Here is the caller graph for this function:

|  |  |  |  |  |  |
| --- | --- | --- | --- | --- | --- |
| void umms.core.annotation.Annotation.setName | ( | String | *name* | ) |  |

Parameters
:   |  |  |
    | --- | --- |
    | name | name of the annotation |

Implemented in umms.core.annotation.BasicAnnotation, and broad.core.annotation.LightweightGenomicAnnotation.

|  |  |  |  |  |  |
| --- | --- | --- | --- | --- | --- |
| void umms.core.annotation.Annotation.setOrientation | ( | char | *orientation* | ) |  |

Implemented in umms.core.annotation.AbstractAnnotation.

|  |  |  |  |  |  |
| --- | --- | --- | --- | --- | --- |
| void umms.core.annotation.Annotation.setOrientation | ( | Strand | *orientation* | ) |  |

Implemented in umms.core.annotation.BasicAnnotation.

|  |  |  |  |  |  |
| --- | --- | --- | --- | --- | --- |
| void umms.core.annotation.Annotation.setOrientedEnd | ( | int | *orientedEnd* | ) |  |

Implemented in umms.core.annotation.BasicAnnotation.

|  |  |  |  |  |  |
| --- | --- | --- | --- | --- | --- |
| void umms.core.annotation.Annotation.setOrientedStart | ( | int | *orientedStart* | ) |  |

Implemented in umms.core.annotation.BasicAnnotation.

|  |  |  |  |  |  |
| --- | --- | --- | --- | --- | --- |
| void umms.core.annotation.Annotation.setReferenceName | ( | String | *refName* | ) |  |

Parameters
:   |  |  |
    | --- | --- |
    | refName | name of the reference sequence / chromosome |

Implemented in umms.core.annotation.BasicAnnotation.

Here is the caller graph for this function:

|  |  |  |  |  |  |
| --- | --- | --- | --- | --- | --- |
| void umms.core.annotation.Annotation.setScore | ( | double | *score* | ) |  |

Implemented in umms.core.annotation.BasicAnnotation, broad.core.annotation.BasicLightweightAnnotation, and broad.core.annotation.LightweightGenomicAnnotation.

|  |  |  |  |  |  |
| --- | --- | --- | --- | --- | --- |
| void umms.core.annotation.Annotation.setStart | ( | int | *start* | ) |  |

Sets a new start position. Will delete and/or truncate blocks if the new start position is greater than the old one. Will extend the first block if the new start position is less than old one.

Parameters
:   |  |  |
    | --- | --- |
    | new | start position (ignoring orientation) of the annotation |

Implemented in umms.core.annotation.BasicAnnotation, and broad.core.annotation.LightweightGenomicAnnotation.

Here is the caller graph for this function:

|  |  |  |  |  |  |
| --- | --- | --- | --- | --- | --- |
| void umms.core.annotation.Annotation.shift | ( | int | *delta* | ) |  |

Shift the positioning of the annotation by a given number of base pairs.

Parameters
:   |  |  |
    | --- | --- |
    | delta | Positive or negative shift to apply to all blocks in the annotation. delta=0  leaves the annotation unchanged. |

Implemented in umms.core.annotation.BasicAnnotation, and umms.core.alignment.AbstractPairedEndAlignment.

|  |  |  |  |  |
| --- | --- | --- | --- | --- |
| int umms.core.annotation.Annotation.size | ( |  | ) |  |

Returns
:   same as length()

Implemented in umms.core.annotation.AbstractAnnotation.

Here is the caller graph for this function:

|  |  |  |  |  |  |
| --- | --- | --- | --- | --- | --- |
| void umms.core.annotation.Annotation.stitchTo | ( | Annotation | *next* | ) |  |

Implemented in umms.core.annotation.BasicAnnotation.

|  |  |  |  |  |
| --- | --- | --- | --- | --- |
| String umms.core.annotation.Annotation.toBED | ( |  | ) |  |

Implemented in umms.core.annotation.Gene, broad.core.annotation.BED, and umms.core.annotation.AbstractAnnotation.

Here is the caller graph for this function:

|  |  |  |  |
| --- | --- | --- | --- |
| String umms.core.annotation.Annotation.toBED | ( | int | *r*, |
|  |  | int | *g*, |
|  |  | int | *b* |
|  | ) |  |  |

Implemented in umms.core.annotation.Gene, broad.core.annotation.BED, and umms.core.annotation.AbstractAnnotation.

|  |  |  |  |  |
| --- | --- | --- | --- | --- |
| String umms.core.annotation.Annotation.toBEDGraph | ( |  | ) |  |

Implemented in umms.core.annotation.AbstractAnnotation.

|  |  |  |  |  |
| --- | --- | --- | --- | --- |
| String umms.core.annotation.Annotation.toCigar | ( |  | ) |  |

Implemented in umms.core.annotation.AbstractAnnotation.

|  |  |  |  |  |
| --- | --- | --- | --- | --- |
| String umms.core.annotation.Annotation.toShortBED | ( |  | ) |  |

Implemented in umms.core.annotation.AbstractAnnotation.

|  |  |  |  |  |
| --- | --- | --- | --- | --- |
| String umms.core.annotation.Annotation.toUCSC | ( |  | ) |  |

Returns
:   chrX:start-end

Implemented in umms.core.annotation.Gene, broad.core.annotation.BasicLightweightAnnotation, umms.core.annotation.AbstractAnnotation, broad.pda.datastructures.Alignments, and broad.core.annotation.LightweightGenomicAnnotation.

Here is the caller graph for this function:

|  |  |  |  |
| --- | --- | --- | --- |
| Annotation umms.core.annotation.Annotation.trim | ( | int | *deltaStart*, |
|  |  | int | *deltaEnd* |
|  | ) |  |  |

Trim annotation by a set number of bases, taking into account blocks.

Parameters
:   |  |  |
    | --- | --- |
    | deltaStart | (Positive) The number of bases to remove from the beginning of gene. e.g. deltaStart=1 removes one base from the front |
    | deltaEnd | (Positive) The number of bases to remove from the end of gene. e.g. deltaStart=1 removes one base fromt he end |

Returns

Implemented in umms.core.annotation.AbstractAnnotation.

|  |  |  |  |  |  |
| --- | --- | --- | --- | --- | --- |
| Annotation umms.core.annotation.Annotation.union | ( | Annotation | *other* | ) |  |

Parameters
:   |  |  |
    | --- | --- |
    | other |  |

Returns
:   union of current instance and other object. If strands agree, then strand is set to that. If the strands conflict, strand is set to UNKNOWN

Implemented in umms.core.annotation.BasicAnnotation.

---

The documentation for this interface was generated from the following file:

- java/umms/core/annotation/Annotation.java


---

Generated on Fri Aug 15 2014 15:11:08 for ESAT by  

 1.8.7
